# Eligibility Classification as a Factor in Understanding Student-Athlete Responses to Collegiate Volleyball Competition

**DOI:** 10.3390/sports9030043

**Published:** 2021-03-23

**Authors:** Rohan Edmonds, Brad Schmidt, Jacob Siedlik

**Affiliations:** 1 Department of Exercise Science and Pre-Health Professions, Creighton University, Omaha, NE 68178, USA; jakesiedlik@creighton.edu; 2 Athletic Performance, Creighton University, Omaha, NE 68178, USA; BradSchmidt@creighton.edu

**Keywords:** athlete monitoring, heart rate variability, freshman, cardiac vagal activity

## Abstract

The current study examined differences in heart rate variability (HRV) across student-athletes of different eligibility classifications and analyzed differences in HRV when competing at home or away. Fourteen female collegiate volleyball players volunteered for the study. Data collection encompassed an entire collegiate season, with comparisons in HRV made between home and away games, as well as pre-gameday, gameday, and post-gameday recordings for the whole squad. Comparisons were also made between student-athlete eligibility classification, with self-reported measures of sleep quality, fatigue, muscle soreness, stress, and mood recorded at the time of HRV measurement. Freshman athletes reported a significantly (*p* < 0.05, η^2^ = 0.17) lower HRV (80.3 ± 9.7) compared to sophomore (85.7 ± 7.2), junior (91.2 ± 8.3), and senior (86.5 ± 7.2) athletes, while junior athletes had a significantly higher HRV when compared to sophomore and senior athletes. All athlete classifications reported similar HRV for home and away games, and there was no difference in HRV for any athlete classification group when comparing pre-gameday, gameday, and post-gameday measures. Freshman athletes reported significantly (*p* < 0.05, η^2^ = 0.23) worse mood states compared to the other eligibility classifications, while self-reported stress was significantly (*p* < 0.05) worse in junior and senior athletes. Results suggest that monitoring the workload of student-athletes based on their eligibility classification holds merit. Collegiate coaching and support staff should be aware of the academic and competitive demands placed on their student-athletes. In particular, freshman athletes adjusting to the increased demands placed on them as collegiate student-athlete may warrant additional support.

## 1. Introduction

Resting heart rate (HR) variability (HRV), the variability between successive heart beats (R-R intervals), is considered a non-invasive indicator of the cardiac autonomic nervous system [1]. An attractive option for testing given its time efficiency and non-invasiveness [2], HRV offers coaches and athletes potential insight into long-term training adaptations, readiness to train, and optimizing training prescriptions [3,4,5]. However, it is important to note that changes in HRV considered undesirable, and potentially indicative of fatigue, may be caused by non-training related factors [6]. Factors such as sleep quality, stress, and mood state have also been shown to influence HRV patterns [7,8,9]. As such, coaches and support staff are encouraged to incorporate subjective measures to better target interventions and enhance training adaptations [6], especially when working with an athletic population that experiences numerous stressors outside of their sporting environment. Research has also shown HRV to be a useful tool for the objective assessment of psychological health and stress across multiple settings [8,10,11,12,13]. As such, HRV may offer value by helping to understand the physiological and psychological demands placed on student-athletes.

Student-athletes are often exposed to myriad stressors from both their sporting and academic commitments [14]. While it must be acknowledged that involvement in sport may serve to alleviate stress [15,16], it has also been shown that athletic participation itself may develop into an added stressor that traditional college students do not encounter [17]. Accompanying the typical stressors associated with collegiate life (academic commitments and family/peer relationships), student-athletes are exposed to unique stressors related to their athletic status [18]. These may include performance-related stressors (i.e., fear of injury, the possibility of being “benched” or red-shirted), or organizational stressors, such as potential conflicts with teammates and/or coaching staff [19]. Furthermore, the loss of “star status” as a high-school athlete, coupled with the increased demands of a collegiate academic workload, may place freshman athletes at an elevated risk. These additional stressors may affect their psychological and physiological wellbeing [18]. While freshman athletes may be at an elevated risk of stress, less is currently known about how student-athletes outside of their freshman year handle the stress of balancing academic and sporting commitments. Earlier research has suggested that senior athletes were less stressed when compared to other collegiate athletes as they had gained experience and learned how to better manage their time [20]. Heller and colleagues [20] observed that senior-eligible female collegiate ice hockey players reported lower stress levels when compared to sophomore and junior eligible athletes.

While it is relatively well known that student-athletes are faced with multiple stressors within their collegiate environment, less is currently known as to how they handle the additional demands of the travel associated with collegiate sport. it is important to recognize that air travel in particular may pose various physiological and psychological stressors that have the potential to influence student-athlete wellbeing [21]. Research has shown that domestic air travel may reduce cardiac vagal modulation, therefore altering cardiac autonomic function [22]. Oliveira-Silva and colleagues [22] noted the reduction in HRV was lesser for individuals with greater physical fitness (greater aerobic capacity and leaner body composition), suggesting that enhanced physical fitness may better equip passengers to cope with the cardiovascular stresses associated with air travel. Indeed, Flatt et al. [6] observed greater reductions in HRV following an international tournament compared to a domestic tournament, despite no change in the physical demands of match play. This would suggest that factors separate from competition, such as the travel associated with competing, may further augment the physiological response to competition [6]. Volleyball is considered a high-intensity intermittent sport with frequent explosive movements [23]. Exercise periods in volleyball are relatively short, lasting roughly nine seconds on average, with periods of rest lasting roughly 12 s interspersed throughout match play and an average work-to-rest ratio ranging between 1:1.6 and 2.2 [24]. The cardiovascular loading during an elite volleyball game is on average 75% HRmax, with large interplayer variability. Given the high number of explosive movements and moderate-to-high cardiovascular load, neuromuscular and cardiovascular fatigue is likely to occur during high-level volleyball games [23].

It could be argued that collegiate athletes, when faced with the potential stresses of their academic and sporting workload, paired with the potential stress of air travel, and lastly, the physiological load of game play, may be susceptible to an increased risk of burnout and potential training maladaptation. Further, given the varying stressors placed on athletes across the eligibility classifications, with freshmen faced with different academic and sporting foci compared with seniors, recognizing how student-athletes respond to the demands placed on them is a key aspect of ensuring their overall health and wellbeing. Understanding how student-athletes across differing years of college eligibility manage the demands of their academic and sporting commitments not only is essential to maximize their ability to compete at a high level but also ensures their overall health and wellbeing are managed appropriately. As such, the primary purpose of this study was to document the differences in HRV across athletes of different eligibility classifications. A secondary purpose of this study was to document any potential differences in HRV when competing at home or away. It was hypothesized that HRV would be similar for athletes across all years of eligibility. Additionally, it was hypothesized that HRV would be lower following away games when compared to home games.

## 2. Materials and Methods

This was a longitudinal study conducted throughout a collegiate volleyball season to document the cardiac autonomic nervous system response to home and away games in a cohort of division I National Collegiate Athletic Association (NCAA) volleyball players. Comparisons were made between home and away games, as well as pre-gameday, gameday, and post-gameday recordings for the whole squad. In addition, HRV was analyzed between athletes in their freshman, sophomore, junior, and senior years of eligibility to identify and potential differences in cardiac autonomic function.

### 2.1. Subjects

Participant characteristics are listed in Table 1. Female volleyball players (n = 14) between 18 and 22 years of age voluntarily enrolled to participate in the study after providing informed consent. Inclusion criteria were defined to ensure that all participants were members of the university’s volleyball program, had competed at the high school level, and had a minimum of two years of competitive volleyball experience. Athletes were assigned according to their collegiate year of eligibility. Before the commencement of the study, ethical approval was obtained from the university institutional review board committee (Study #2000381). All athletes were informed of the possible risks involved, advised that they could withdraw at any stage of the data collection period, and provided written informed consent before participating in any aspect of the study.

### 2.2. Procedures

Data collection encompassed 30 games (16 home and 14 away), commencing in late August, prior to a pre-season invitational tournament, and concluding after the team’s final game of the season at the Women’s NCAA Volleyball Tournament in early December. The typical training week consisted of two weightlifting sessions and three on-court practices, with a more detailed description shown in Table 2. Before the commencement of data collection, skinfold thickness was measured to ascertain body fat percentage. Measurements were recorded to the nearest 0.5 mm at three anatomical landmarks (triceps, iliac, and thigh) on the right side of the body.

In line with previously established guidelines [5], daily HR and HRV measures were performed each morning after waking and bladder emptying. A 60 s (5 s stabilization and 55 s recording) HR and HRV (lnRMSSD) measurement was recorded in the seated position via the use of an infrared pulse finger sensor (ithlete; HRV Fit Ltd. Southampton, UK) connected to a smartphone application (ithlete HRV), as previously validated [24]. Athletes were instructed to follow the paced breathing (7.5 breaths per minute) within the app to ensure consistency of measurement. Any ectopic beats or artifacts were removed by an in-built algorithm, with the lnRMSSD value multiplied by 20 for easier interpretation, providing a ~100-point scale for analysis. Using a visual analog scale (1 = worst rating and 9 = best rating), athletes self-reported measures of sleep quality, fatigue, muscle soreness, stress, and mood after the HR and HRV measurement. Each recording was then assigned based on location (home or away) and the day (pre-gameday, gameday, post-gameday) of recording.

### 2.3. Statistical Analyses

Data were analyzed using JASP (JASP Team, Version 0.14.1, Amsterdam, The Netherlands), with data presented as means ± standard deviation. Data normality was assessed using a Shapiro–Wilk test of normality. If the data met the normality assumption, differences between recordings days (pre-gameday vs. gameday vs. post-gameday) and location (home vs. away) for the entire team, and each class (freshmen, sophomore, junior, and senior) were identified using a simple one-way analysis of variance (ANOVA). Differences in HR, HRV, and the self-reported measures between athlete eligibility classifications were also identified using a simple one-way ANOVA, with the partial eta-squared (η^2^) used to estimate the effect size. Where significance was observed, a Tukey post-hoc test was performed. A Spearman’s rank correlation coefficient was used to identify relationships between HR, HRV, and the self-reported measures of sleep quality, fatigue, muscle soreness, stress, and mood. An alpha level of *p* < 0.05 was set for all statistical analyses, with the threshold values for both η^2^ established as small (0.02), moderate (0.13), and large (0.26) [25].

## 3. Results

Athletes in the different eligibility classifications were of similar (*p* > 0.05) height, weight, and body fat percentage (Table 1).

**Figure 1:**
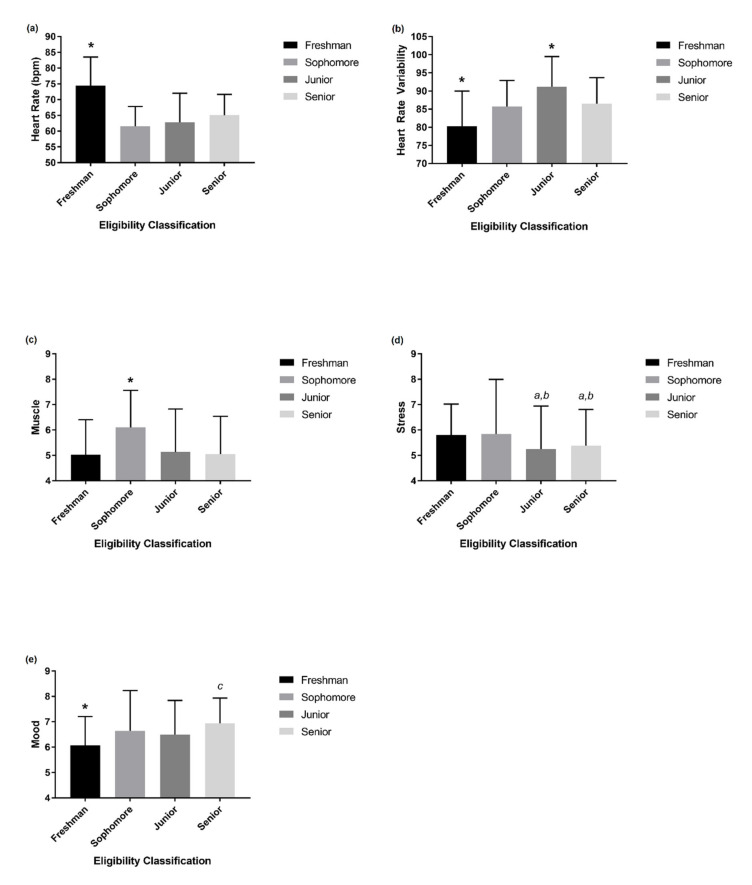
Mean (± standard deviation) heart rate (**a**), heart rate variability (**b**), and athlete self-reported measures of muscle soreness (**c**), stress (**d**), and mood state (**e**) across student-athlete eligibility classifications during the collegiate season. * *p* < 0.05 compared to all other eligibility classifications, # *p* < 0.05 compared to sophomore and senior eligibility classifications, *a p* < 0.05 compared to freshman athletes, *b p* < 0.05 compared to sophomore athletes, *c p* < 0.05 compared to sophomore and junior athletes.

On average, freshman athletes exhibited significantly (*p* < 0.05, η^2^ = 0.26) higher HR (74.4 ± 9.1 bpm) compared to sophomore (61.5 ± 6.3 bpm), junior (62.9 ± 9.2 bpm), and senior (65.1 ± 6.6 bpm) athletes over the course of the season (Figure 1a).

Freshman athletes also had a significantly (*p* < 0.05, η^2^ = 0.17) lower HRV score (80.3 ± 9.7) compared to sophomore (85.7 ± 7.2), junior (91.2 ± 8.3), and senior (86.5 ± 7.2) athletes over the volleyball season (Figure 1b). Furthermore, junior athletes had a significantly higher HRV score when compared to sophomore and senior athletes (Figure 1b).

All athlete classifications reported similar (*p* > 0.05) HR and HRV scores for home and away games. Likewise, there was no difference (*p* > 0.05) in HR or HRV scores for any athlete classification group when comparing pre-gameday, gameday, and post-gameday recordings.

Freshman (5.0 ± 1.4), junior (5.1 ± 1.7), and senior (5.0 ± 1.5) classified athletes reported similar (*p* > 0.05) measures of muscle soreness, while all were significantly lower (*p* < 0.05, η^2^ = 0.18) when compared to sophomore (6.1 ± 1.5) athletes (Figure 1c). Self-reported levels of stress were significantly (*p* < 0.05) worse in junior (5.2 ± 1.7) and senior (5.3 ± 1.4) athletes when compared to freshman (5.8 ± 1.2) and sophomore (5.8 ± 2.1) athletes (Figure 1d). Freshman (6.0 ± 1.1) athletes reported a significantly (*p* < 0.05, η^2^ = 0.23) lower mood state compared to sophomore (6.6 ± 1.6), junior (6.5 ± 1.3), and senior (6.9 ± 1.0) athletes (Figure 1e). In contrast, senior athletes reported a significantly (*p* < 0.05, η^2^ = 0.21) higher mood state compared to sophomore and junior athletes (Figure 1e). Self-reported measures of sleep and fatigue were similar (*p* > 0.05) across all athlete classifications.

Freshmen reported similar (*p* > 0.05) measures of sleep, muscle soreness, and stress when comparing home and away games. Freshmen reported significantly (*p* < 0.05) worse measures of fatigue when away (5.5 ± 1.4) compared to at home (6.3 ± 1.2) (Table 3). Likewise, freshmen also reported significantly (*p* < 0.05) worse mood state for away games (5.8 ± 0.8) compared to home games (6.3 ± 1.3) (Table 3). There was no difference in pre-gameday, gameday, and post-gameday self-reported measures for freshman athletes.

There were no differences in any of the self-reported measures when at home or away for sophomore athletes. Self-reported measures of sleep, stress, and mood were similar when comparing pre-gameday, gameday, and post-gameday self-reported measures for sophomore athletes. However, sophomore athletes reported significantly (*p* < 0.05) worse fatigue post-gameday (5.0 ± 1.5) compared to pre-gameday (5.7 ± 1.3) and gameday (6.0 ± 1.5). Sophomores also reported significantly (*p* < 0.05) worse muscle soreness pre-gameday (5.6 ± 1.3) compared to gameday (6.4 ± 1.3) and post-gameday (6.7 ± 1.5).

Juniors reported similar values for all self-reported measures at home and when away. Likewise, there was no difference in pre-gameday, gameday, and post-gameday self-reported measures for junior athletes.

Seniors reported similar (*p* > 0.05) measures of sleep, fatigue, stress, and mood state when comparing home and away games. However, muscle soreness was significantly (*p* < 0.05) worse when away (4.6 ± 1.4) compared to at home (5.2 ± 1.5) (Table 3). There was no difference in pre-gameday, gameday, and post-gameday self-reported measures for senior athletes.

There were no significant correlations observed between HR or HRV and any self-reported measures for freshman athletes.

Significant positive relationships were observed between HRV and sleep (*p* = < 0.001, rho = 0.415), HRV and fatigue (*p* = < 0.001, rho = 0.316), HRV and mood state (*p* = < 0.001, rho = 0.286), and HRV and stress (*p* = 0.023, rho = 0.183) for sophomore athletes.

Significant negative relationships were observed between HRV and muscle soreness (*p* = 0.002, rho = −0.264) and HRV and mood state (*p* = 0.004, rho = −0.237) for junior athletes. No other significant correlations were reported between HR or HRV and sleep, fatigue, or stress for the junior athletes.

Significant positive relationships were reported between HRV and fatigue (*p* = < 0.001, rho = 0.354) and HRV and muscle soreness (*p* = < 0.001, rho = 0.340) for senior athletes. No other significant relationships were observed between HR or HRV and sleep, stress, or mood state for senior athletes.

## 4. Discussion

The primary purpose of this study was to document the differences in HRV across student-athletes of different eligibility classifications. A secondary purpose of the current study was to identify any potential differences in HRV when competing at home or away. The novel finding from this study was that student-athletes in their freshman year exhibited significantly lower HRV throughout the collegiate season when compared against sophomore, junior, and senior student-athletes. Likewise, junior student-athletes reported significantly higher HRV during the season compared to sophomore and senior student-athletes. Another noteworthy observation from this study was the similarity in HRV across home and away games, with no significant differences in HRV reported the day before, the day of, or the day after games played at home or away.

While numerous studies have examined HRV across various collegiate sporting environments [26,27,28,29,30], to date, no studies have documented the differences in HRV across eligibility classifications. Indeed, while earlier research examining the HRV responses of collegiate football players shows the value of documenting cardiac parasympathetic activity based on position [29], results from the current study suggest the additional benefit of monitoring HRV based on student-athlete eligibility. In particular, freshman student-athletes reported the lowest HRV and lowest self-reported mood state throughout the season, while the self-reported measures of sleep and fatigue were similar across all classifications. Given the similar HRV responses for home and away games across all eligibility classifications, paired with similar self-reported fatigue, these results suggest that factors outside of training and competition may contribute to the reduction in HRV observed in the freshman student-athletes. Freshman athletes adjusting to their potential loss of playing time or “star status” may lead to a reduced mood state [18]. This reduced mood state, paired with a potential loss of playing time, may lead to a reduction in HRV. Indeed, research has shown that a reduction in cardiac parasympathetic activity is predictive of negative mood states after the deprivation of usual exercise activities [31,32]. In a cohort of physically active adults, Weinstein et al. [32] found that exercise withdrawal resulted in a significantly higher negative mood state in physically active individuals when compared against a control group who were able to maintain their regular aerobic exercise habits. This increase in negative mood state was preceded by a reduction in cardiac vagal modulation. More recently, Deo and colleagues [33] also highlighted that changes in HRV reflect changes in mood state, with a reduction in cardiac parasympathetic activity associated with negative mood states in daily life. Freshman athletes adjusting to the potential reduction in playing time coupled with the possible loss of “star status” achieved while competing in high school may increase their susceptibility to a negative or depressive mood state. This in turn may lead to a reduction in HRV, potentially increasing their susceptibility to burnout and limiting their ability to compete at their peak. The inclusion of mindfulness practice, a technique that may encourage wellbeing and has been shown to improve HRV and reduce self-reported stress [34], may offer benefit within a collegiate sporting environment. While the inclusion of additional recovery days may not be viable, coaches and support staff may find benefit in incorporating aspects of mindfulness practice to help to encourage positive wellbeing, while also improving cardiac autonomic balance.

One noteworthy observation from the current study was the lack of change in HRV between pre-gameday, gameday, and post-gameday measures, following games played at home and away. Indeed, it has been well documented that higher exercise intensity is associated with a reduction in cardiac parasympathetic recovery [35,36,37,38,39]. In particular, a recent review by Stanley et al. [39] suggests that 24−48 h are needed to fully restore cardiac autonomic balance following threshold-intensity exercise, and at least 48 h following high-intensity aerobic exercise. Given volleyball is considered a high-intensity intermittent sport, with the average cardiovascular load during an elite volleyball game roughly 75% HRmax [23], it would be anticipated that athletes in the current study exhibit a reduction in HRV the morning after a game. However, research has also shown that recovery to within resting levels of HRV is quicker in highly trained individuals [39], suggesting that athletes in the current study were physiologically well equipped to manage the cardiovascular demands of games played at home and away. While the lack of a reduction in HRV is somewhat surprising, there was no difference in HRV between home and away games, suggesting the travel for away games had no perceptible influence on cardiac autonomic regulation. This result supports the previous observations of Oliveira-Silva et al. [22] that indicate passengers with enhanced physical fitness can better handle the potential cardiovascular stresses of air travel.

Of note, while there was no apparent change in HRV, subjective measures of mood state, fatigue, and muscle soreness were worse when away compared to home. This would suggest that, while athletes in the current study were physiologically able to cope with the demands of away games, they were subjectively perceived to be more demanding. Further, the various athlete self-reported measures also differed based on eligibility classification, with freshman athletes reporting worse measures of fatigue and mood state for away games, while seniors reported worse muscle soreness for away games. While the physiological response to away games was similar, these results indicate that freshman athletes experience a heavier psychological response to competing away. A loss of playing time or “star status” on a collegiate roster may elicit a greater response for freshman athletes while on the road. As such, coaches and support staff may benefit from better understanding the psychological influence of away games and incorporating evidence-based interventions of such mindfulness practices specific to the various athletic eligibility classifications.

This study, while reflective of a typical collegiate volleyball squad, was limited by the small sample size. Future studies encompassing more collegiate athletes across multiple sports and tracking both male and female athletes may offer a more definitive understanding and further support the current findings. Further research is also warranted within the collegiate student-athlete population to help to identify whether the reduced HRV profile of freshman athletes was a result of the reduced self-reported mood state and/or fatigue, or whether the reduction in HRV preceded the diminished mood state and heightened fatigue. Identifying whether HRV is predictive of a reduced mood state may allow coaches and support staff to better manage student-athlete wellbeing, while proactively incorporating practices to encourage mindfulness may limit any detrimental influence of the sporting and academic demands placed on student-athletes.

## 5. Conclusions

Based on results from the current study, monitoring the workload of student-athletes based on their eligibility classification holds merit. University athletic departments and collegiate coaching and support staff should be cognizant of both the academic and competitive demands placed on their student-athletes. In particular, freshman athletes who are adjusting to the increased demands placed on them in their new capacity as collegiate student-athletes warrant additional support. Results from the current study also add further support to the use of subjective wellness indices, in conjunction with HRV, among collegiate athletes, as they may allow for more targeted interventions to better support student-athlete health and wellbeing.

## Figures and Tables

**Table 1:** Participant characteristics and anthropometric measurements.

Anthropometric Measure	Squad (n = 14)	Freshmen (n = 3)	Sophomores (n = 3)	Juniors (n = 4)	Seniors (n = 4)
Age (years)	20.4 ± 1.5	18.0 ± 0.0	20.0 ± 0.0	21.0 ± 0.8	21.8 ± 0.5
Height (cm)	182.5 ± 8.5	187.1 ± 3.9	180.3 ± 11.1	178.4 ± 5.2	184.8 ± 11.6
Body Mass (kg)	77.6 ± 7.9	75.3 ± 5.2	75.6 ± 14.3	79.6 ± 6.5	79.0 ± 7.8
Body Fat (%)	25.6 ± 2.9	22.9 ± 1.9	25.7 ± 2.6	27.1 ± 4.0	26.1 ± 2.0

**Table 2:** Weekly training schedule for the women’s volleyball team during the collegiate season.

Day	Home Game	Away Game
Monday	Off Day	Off Day
Tuesday	–	Pre-practice weightlifting (30–45 min) and Practice (120–150 min)
Wednesday	–	Pre-practice weightlifting (30–45 min) and Practice (120–150 min)
Thursday	–	Practice (90–120 min)
Friday	–	Game Day
Saturday	Recovery Day	Game Day Film, walkthrough and active recovery (30–60 min)
Sunday	Game Day	Travel Day

**Table 3:** Mean (± standard deviation) heart rate, heart rate variability, and athlete self-reported measures across student-athlete eligibility classifications for home and away games during the collegiate season.

Variable	Freshman (n = 3)		Sophomore (n = 3)		Junior (n = 4)		Senior (n = 4)	
	Home	Away	Home	Away	Home	Away	Home	Away
Heart Rate (bpm)	72.6 ± 9.9	66.9 ± 9.7	72.7 ± 7.7	69.3 ± 8.1	60.8 ± 6.1	66.5 ± 7.3	62.1 ± 6.4	64.9 ± 6.7
Heart Rate Variability	78.4 ± 9.9	81.6 ± 8.7	85.9 ± 7.9	85.8 ± 6.5	91.6 ± 8.7	90.1 ± 7.8	84.7 ± 8.2	87.4 ± 8.5
Sleep (VAS 1-9)	6.1 ± 1.2	6.0 ± 1.5	6.2 ± 1.2	6.2 ± 1.4	6.2 ± 2.1	6.3 ± 1.8	6.4 ± 1.9	6.3 ± 1.7
Fatigue (VAS 1-9)	6.3 ± 1.2	5.5 ± 1.4 *	6.0 ± 1.3	5.7 ± 1.4	5.9 ± 1.5	5.8 ± 1.4	5.5 ± 1.5	5.4 ± 1.5
Muscle Soreness (VAS 1-9)	5.2 ± 1.3	5.1 ± 1.8	4.9 ± 1.4	6.1 ± 1.5	6. 1 ± 1.5	5.2 ± 1.5	6.1 ± 1.4	4.6 ± 1.4
Stress (VAS 1-9)	5.8 ± 1.2	5.4 ± 1.7	5.8 ± 1.3	5.1 ± 1.6	5.6. ± 2.2	5.3 ± 1.5	6.0 ± 2.1	5.5 ± 1.3
Mood (VAS 1-9)	6.3 ± 1.3	5.8 ± 0.8	5.8 ± 1.3	6.3 ± 1.3	6.6 ± 1.6	6.8 ± 1.2	6.6 ± 1.6	6.8 ± 1.2

* *p* < 0.05 compared to home games.

## Data Availability

The data presented in this study are available on request from the corresponding author. The data are not publicly available due to IRB imposed restrictions.
